# Trends in HIV Testing Among Adults in the Deep South: Behavioral Risk Factor Surveillance System, 2017–2023

**DOI:** 10.1007/s10461-025-04776-x

**Published:** 2025-06-17

**Authors:** Precious Patrick Edet, Azad R. Bhuiyan, Trisha Arnold, Amy Nunn, Andrew Yockey, Ruaa Al Juboori, Hannah K. Allen

**Affiliations:** 1 Department of Public Health, University of Mississippi, University, MS 38677, USA; 2 William Magee Institute for Student Wellbeing, University of Mississippi, University, MS 38677, USA; 3 Department of Epidemiology and Biostatistics, Jackson State University, Jackson, MS 39213, USA; 4 Department of Psychiatry and Human Behavior, Brown University Health, Providence, RI 02912, USA; 5 Department of Behavioral and Social Health Sciences, Brown University, Providence, RI 02912, USA

**Keywords:** HIV testing, Deep south, BRFSS, Trend analysis

## Abstract

HIV testing is an entry point for both HIV prevention and treatment, and the CDC recommends that all adults of reproductive age undergo HIV testing at least once in their lifetime. However, HIV testing rates remain suboptimal. This study analyzed trends in HIV testing using Behavioral Risk Factor Surveillance System data from 2017 to 2023 across nine Deep South states—Alabama, Florida, Georgia, Louisiana, Mississippi, North Carolina, South Carolina, Tennessee, and Texas. Descriptive statistics and Joinpoint linear regression were employed to assess lifetime HIV testing and testing within the past 12 months. Findings showed that the overall trend in having ever tested for HIV rose from 43% in 2017 to 47% in 2019 but declined to 40% in 2022, with a slight increase to 41% in 2023. Significant declines in ever testing were observed in North Carolina and among adults aged 25–44, non-Hispanic Black, non-Hispanic multiracial, and those identifying as lesbian or gay. Furthermore, the overall trend in HIV testing in the past 12 months declined significantly from 48% in 2017 to 42% in 2022, with a slight increase to 43% in 2023. Significant declines were found in Florida, Georgia, Louisiana, North Carolina, and South Carolina, and among adults aged 18–34, non-Hispanic White, non-Hispanic Black, male, female, and heterosexual individuals. These trends, observed largely during the COVID-19 pandemic, underscore the need to scale up HIV prevention and care initiatives, particularly in populations and regions experiencing significant declines. Trends should continue to be monitored and examined post-COVID pandemic.

## Introduction

In 2022, approximately 1.2 million people aged 13 or older were living with HIV knowingly or unknowingly in the United States (U.S.) [1, 2]. Despite a 12% decrease in new HIV infections from 2018 to 2022, more than 38,000 people received an HIV diagnosis in 2022 [1, 3, 4]. The Southern region of the United States has consistently faced the highest HIV burden, accounting for more than half (51–53%) of all HIV infections in the U.S [3, 5-7]. Within this region, the Deep South—Alabama, Florida, Georgia, Louisiana, Mississippi, North Carolina, South Carolina, Tennessee, and Texas, has been particularly impacted, with an HIV diagnosis rate as high as 65% [8].

The federal initiative “Ending the HIV Epidemic in the U.S.” (EHE) by the U.S. Department of Health and Human Services (DHHS) aims to reduce new HIV infections in the United States by 75% and 90% by 2025 and 2030, respectively [9]. Additionally, Healthy People 2030 goals set by the Office of Disease Prevention and Health Promotion within the DHHS emphasizes increasing the proportion of people who are aware of their HIV status through testing and reducing the number of new HIV infections by 2030 [10].

HIV testing is the entryway for HIV prevention and treatment [11]. The Centers for Disease Control and Prevention (CDC) report and several studies have confirmed that timely HIV testing and treatment reduce transmission, particularly when combined with strategies like pre-exposure prophylaxis (PrEP) [12-14]. The CDC recommends that all individuals aged 13 to 64 years should get tested for HIV at least once in their lifetime, and at-risk individuals, including racial and sexual minorities and people who inject drugs, should get tested more often—at least once a year [12, 15]. However, despite these recommendations and ongoing prevention efforts, HIV testing rates are suboptimal, particularly among people of color (57–60%) who are at highest risk of HIV acquisition [11, 16-19].

Several studies have investigated HIV testing trends across the U.S. over time. Ansa and colleagues [20] used statewide data for Georgia and revealed a slight, although not significant, decline in ever testing for HIV (46% in 2011 to 44% in 2015). Moreover, another study by Patel and colleagues [21] demonstrated a significant increase in ever testing for HIV (43% in 2011 to 46% in 2017), and the rate of testing within the past 12 months increased from 13 to 15% during the same period. Despite these upward trends, Patel et al. [21] highlighted that fewer than half of U.S. adults had ever tested for HIV during the study period, even after a decade since CDC’s recommendations on HIV testing were published. Additionally, the CDC [4] reported an increase in HIV testing across the U.S. in 2022, adding that a higher percentage of people with HIV were aware of their status in 2022 than in 2018, with a slight increase from 86 to 87%.

The Southern U.S. is identified as a priority region in the EHE plan, given that it accounts for more than half of all new HIV diagnoses nationally [3, 5-7]. However, the Deep South continues to have poorer HIV outcomes compared to other Southern States [8, 22], with 20 counties from this region listed as priority areas in the EHE plan [9]. According to Reif et al. [23], the rate of HIV diagnoses in the Deep South states was 24 per 100,000 people in 2014, which was 45% higher than the rate in other Southern states (Arkansas, Delaware, District of Columbia, Kentucky, Maryland, Oklahoma, Virginia, and West Virginia), which had a combined diagnosis rate of 17 per 100,000 people, aligning with the national average. Examining trends in HIV testing in the Deep South is critical for ultimately reducing transmission rates.

This study had two aims: (1) examine trends in ever been tested for HIV (i.e., a person has ever been tested at some time in their life) among adults in the Deep South; and (2) examine HIV testing in the past 12 months among adults in the Deep South, utilizing the 2017–2023 Behavioral Risk Factor Surveillance System (BRFSS) data. This study will identify specific demographic groups with potential recent declines in HIV testing who may benefit from targeted intervention efforts. Findings from this study will also contribute to monitoring progress toward achieving national EHE and Healthy People 2030 goals and may help inform public health interventions, thereby contributing to the broader goal of preventing HIV spread and ending the HIV epidemic in the United States.

## Methods

### Study Design and Participants

This study utilizes cross-sectional study data from the 2017–2023 BRFSS to investigate trends in ever been tested for HIV and HIV testing in the past 12 months in the Deep South region of the United States—Alabama [AL], Florida [FL], Georgia [GA], Louisiana [LA], Mississippi [MS], North Carolina [NC], South Carolina [SC], Tennessee [TN], and Texas [TX], as defined in previous studies [8, 23]. While each year’s data represents a cross-sectional snapshot, the multi-year analysis allows for the assessment of HIV testing trends over time. Participants were at least 18 years old, non-institutionalized, resided in the Deep South, and had access to either a landline or cell phone. The state of Florida did not collect BRFSS data in 2021.

### Data Source

This study utilized secondary data from the 2017–2023 BRFSS datasets with selected cases from states in the Deep South. The BRFSS dataset is a nationally representative dataset administered annually by the CDC across all 50 U.S. states, District of Columbia, and U.S. territories including Guam, Puerto Rico, and the Virgin Islands [24]. The BRFSS dataset utilizes a complex sampling methodology to ensure that data collected is generalizable to the general population [24]. To accommodate the complex sampling design, the BRFSS data was weighted, clustered, and stratified so that findings are generalizable to the entire population. The BRFSS collects data on health risk behaviors, preventive services and chronic health conditions from a representative sample of noninstitutionalized adults aged 18 years or older residing in the United States and territories [24]. Data were collected through landline and cellular telephones by means of a Random-Digit Dialing [24].

Weighting procedures employed by BRFSS changed in 2011 when it began using a new methodology known as iterative proportional fitting, also known as “raking” [24]. This process utilized data from cellular telephone surveys allowing for the addition of three demographic characteristics (education, marital status, home renter/owner) to age, race/ethnicity, and sex, to reduce biases associated with low survey coverage of people with certain demographic characteristics [24]. These additional characteristics made the BRFSS sample more representative and generalizable with respect to individual states’ populations based on the largest possible cross-section of demographic characteristics [24].

### Measures

#### Independent Variables

The independent variables were sociodemographic factors including state (AL, FL, GA, LA, MS, NC, SC, TN, and TX), age group (18–24, 25–34, 35–44, 45–54, 55–64, and 65 or older), race/ethnicity (non-Hispanic [NH] White, NH Black, NH other race, NH multiracial, and Hispanic), sex (male and female), sexual orientation (lesbian or gay, hetero-sexual, bisexual, and other), education level (less than high school, high school, some college, and college graduate), annual income level in U.S. Dollars (<$15k, $15k–<$25k, $25k–<$35k, $35k–<$50k, and $50k or more), employment status (employed for wages, self-employed, unemployed, and retired), and marital status (not married, married, divorced, and widowed), which provided information on the demographic characteristics of the study population. These variables were also used to estimate the percentage of ever been tested for HIV and testing for HIV in the past 12 months among adults in the Deep South.

#### Dependent Variables

The dependent variables were HIV testing (ever) and HIV testing in the past 12 months. To obtain data on ever testing for HIV, participants were asked, “Have you ever been tested for HIV?” Responses included “yes”, “no”, “don’t know” and “refused”. To obtain data on HIV testing in the past 12 months, only participants who reported ever testing for HIV were asked, “In what month and year was your last HIV test?” HIV tests conducted at any time in the preceding year beginning in January (12 months from the start of each interview year) through December of the current interview year (end of each interview year) were coded as tests conducted in the past 12 months from the date of interview in each given year. Responses were then dichotomized into two categories: “last HIV testing done in the past 12 months” and “last HIV testing done in more than 12 months.”

All “don’t know,” “refused,” and missing responses were excluded from the analysis to minimize the underestimation of HIV testing and data accuracy, and to be consistent with similar prior analyses [20, 21, 25-27].

### Data Analysis

Prior to analyzing the data, the BRFSS dataset was weighted, clustered, and stratified using _LLWPWT (Final Weight), _PSU (Primary Sampling Unit), and _STRTR (Stratification) variables, respectively, to ensure that the data was accurately analyzed so findings were generalizable to the target population. Descriptive statistics of dependent and independent variables were then performed for each year using Proc surveyfreq procedures in SAS. Weighted frequencies, unweighted count, and 95% confidence intervals (95% CI) provided descriptive statistical information about the study population.

To examine trends, we used descriptive statistics to calculate estimated percentages and their respective standard errors of ever been tested for HIV and testing for HIV in the past 12 months among adults in the Deep South, overall, and by state, age group, race/ethnicity, sex, sexual orientation, education level, annual income level (USD), employment status, and marital status, using Proc surveyfreq procedures in SAS.

Additionally, we performed Joinpoint linear regression to determine the Annual Percent Change (APC) and Average Annual Percent Change (AAPC) for each trend observed during the study period. An Excel spreadsheet comprising each given year of interview, estimated weighted percentages of ever been tested for HIV or HIV testing in the past 12 months by sociodemographic factors for the corresponding year, and standard errors of weighted percentages were used to create the Joinpoint input file which was then uploaded to the Joinpoint software. Permutation test was employed to identify the number of join points, while a parametric method was utilized to calculate the 95% CI for the APC and AAPC for overall trends [28]. Significant changes were identified as shifts in the rate of increase or decrease in the AAPC, as indicated by a 95% CI which did not include 1. The assumptions for Joinpoint linear regression were assessed and validated, ensuring the accuracy of the results.

SAS Institute, Incorporated, statistical package version 9.4 [29] and Joinpoint software version 5.1.0 (Available online: https://surveillance.cancer.gov/joinpoint/) [28, 30] were used to perform the data analyses for this study.

## Results

### Sociodemographic Characteristics of the Study Population

The BRFSS datasets from 2017 to 2023 consisting of data from the Deep South region (AL, FL, GA, LA, MS, NC, SC, TN, and TX) comprised 476,439 respondents, representing 510,762,597 adults in the region. During the study period, 153,260 (42%) respondents reported ever been tested for HIV while 44,330 (45%) respondents reported testing for HIV in the past 12 months. The majority of respondents were NH White (55%, *n* = 312,692), 65 years or older (22%, *n* = 175,006), female (52%, *n* = 266,020), and heterosexual (93%, *n* = 161,822). Additionally, respondents were predominantly employed for wages (46%, *n* = 178,364), had some college education (31%, *n* = 130,998), married (47%, *n* = 215,392), and earned $50,000 or more annually (48%, *n* = 165,187). Table 1 provides the demographic characteristics of respondents in detail.

### Trends in Ever Been Tested for HIV Among Adults in the Deep South from 2017 to 2023

In the Deep South, the percentage of adults ever undergoing HIV testing increased from 43% in 2017 to 47% in 2019 (APC: 2.6; 95% CI: − 12.4, 20.1), then decreased to 40% in 2022, followed by a slight increase to 41% in 2023 (APC: − 3.1; 95% CI: − 8.0, 2.0). Though the overall trend indicated a decrease, it was not statistically significant (AAPC: − 1.3; 95% CI: − 4.0, 1.6). Additionally, all states in the Deep South, except LA, experienced a decrease in the percentage of ever been tested for HIV among adults. However, only NC experienced a significant decrease from 45% in 2017 to 40% in 2023 (AAPC: − 3.0; 95% CI: − 5.7, − 0.2). Findings also revealed a slight decrease in ever been tested for HIV from 36 to 31% among 18–24-year-olds (AAPC: − 4.7; 95% CI: − 9.7, 0.7), and significant decreases from 59 to 51% and 63–57% among 25–34-year-olds (AAPC: − 3.6; 95% CI: − 6.5, − 0.6) and 35–44-year-olds (AAPC: − 2.3; 95% CI: − 4.0, − 0.5), respectively, during the same period. In contrast, there were increased trends among adults aged 45 or older, although not statistically significant.

Furthermore, there were decreases in ever been tested for HIV among all racial/ethnic groups during the study period, except among NH White adults. However, a significant decrease was only observed among NH Black (AAPC:− 2.4; 95% CI: − 4.4, − 0.4) and NH multiracial adults (AAPC:−2.9; 95% CI: − 5.5, − 0.1). Similarly, there was a significant decrease in ever been tested for HIV among respondents who identified as lesbian or gay from 72% in 2017 to 51% in 2023 (AAPC: − 6.7; 95% CI: − 10.3, − 3.0). However, the percentage decrease observed among heterosexuals, bisexual, or “other” adults was not significant. Among sexes, a decreased trend was observed in both males (AAPC: − 1.5; 95% CI: − 3.6, 0.5) and females (AAPC: − 1.5, 95% CI: − 4.2, 1.2), though this trend was not statistically significant.

The percentage of ever been tested for HIV also decreased across all education and income levels from 2017 to 2023, with a significant decrease observed only among college graduates (AAPC: − 2.1; 95% CI: − 4.0, − 0.0). Across various employment and marital status categories, a decrease in ever testing for HIV was mostly observed. However, during the same period, increased trends from 20 to 24% and 52–53% were observed among retired (AAPC: 2.0; 95% CI: − 0.8, 4.8) and divorced persons (AAPC: 3.6; 95% CI: − 3.4, 11.0), respectively, although not statistically significant.

**Fig. 1:**
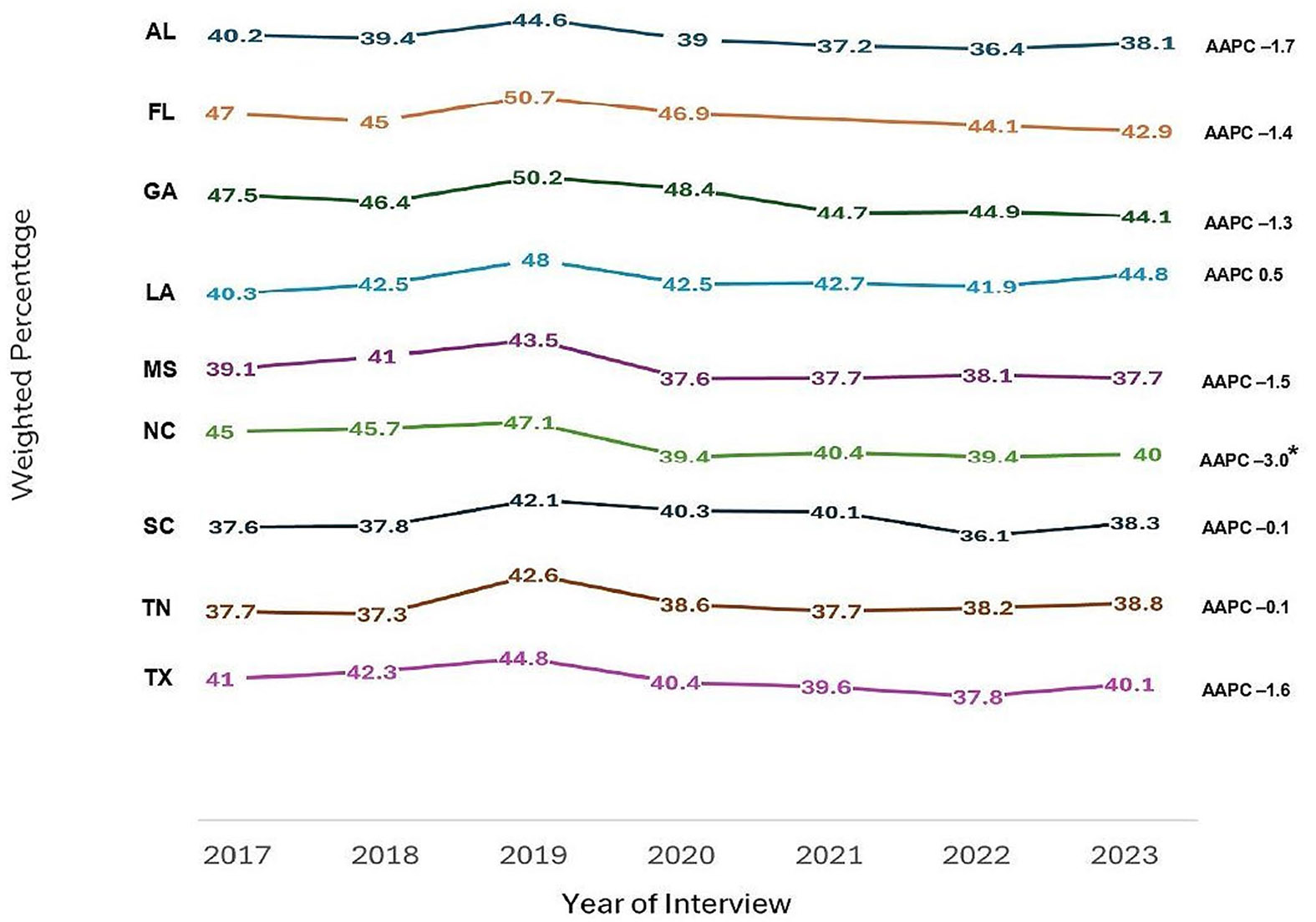
Trends in ever been tested for HIV by state among adults in the deep south, behavioral risk factor surveillance system, 2017–2023. The Deep South is defined as Alabama [AL], Florida [FL], Georgia [GA], Louisiana [LA], Mississippi [MS], North Carolina [NC], South Carolina [SC], Tennessee [TN], and Texas [TX]. *Denotes statistical significance (Average Annual Percent Change [AAPC] 95% confidence interval does not include 1)

Table 2 presents the weighted percentages of ever been tested for HIV by year of interview, along with the AAPCs and corresponding 95% CIs of each variable. Figure 1 illustrates the graphical depiction of overall trends in ever been tested for HIV by state among adults in the Deep South from 2017 to 2023.

### Trends in HIV Testing in the Past 12 Months Among Adults in the Deep South from 2017 to 2023

The percentage of testing for HIV in the past 12 months among adults decreased from 48% in 2017 to 47% in 2019 (APC: − 1.6; 95% CI: − 9.8, 7.4), followed by a further decrease to 42% in 2022 and a slight increase to 43% in 2023 (APC: − 2.8; 95% CI: − 6.0, 0.6), with the overall decline being significant (AAPC: − 2.4; 95% CI: − 4.0, − 0.7). Additionally, all states in the Deep South experienced a decrease in HIV testing within the past 12 months, except for AL which experienced a slight increase (AAPC: 0.1; 95% CI: − 2.5, 2.8). However, significant declines were observed only in FL (AAPC: − 3.5; 95% CI: − 4.8, − 2.2), GA (AAPC: − 4.3; 95% CI: − 6.2, − 2.5), LA (AAPC: − 2.6; 95% CI: − 4.5, − 0.7), NC (AAPC: − 2.8; 95% CI: − 4.8, − 0.9), and SC (AAPC: − 4.0, 95% CI: − 6.3, − 1.7).

Across age groups, there were significant declines in HIV testing within the past 12 months from 2017 to 2023, except among individuals aged 35–44 (AAPC: − 0.7; 95% CI: − 1.2, 2.6). There were also significant decreases among NH White and NH Black individuals from 38 to 34% (AAPC: − 3.0; 95% CI: − 4.3, − 1.7) and 66–54% (AAPC: − 3.5, 95% CI: − 4.3, − 2.7), respectively. Additionally, there were decreases among other racial groups, although not statistically significant.

Furthermore, from 2017 to 2023, there were significant decreases in HIV testing in the past 12 months among males (AAPC: − 2.6; 95% CI: − 4.0, − 1.2) and females (AAPC: − 2.2; 95% CI: − 3.0, − 1.5). Decreased trends were also observed across all sexual identities; however, a significant decrease was only observed among heterosexual adults from 47% in 2017 to 39% in 2023 (AAPC: − 2.6; 95% CI: − 3.9, − 1.2).

Among education levels, the only significant decline was among adults with some college education, with testing rates decreasing from 51% in 2017 to 44% in 2023 (AAPC: − 3.0; 95% CI: − 4.7, − 1.3). Additionally, there were significant decreases observed among respondents earning an annual income of less than $35,000 compared to their counterparts earning higher. Significant decreases were also observed across all employment categories except for respondents who were self-employed, where the decrease observed was not significant (AAPC: − 0.9; 95% CI: − 4.6, 2.9). Among marital status categories, decreased trends in HIV testing within the past 12 months were observed; however, these decreases were not statistically significant.

**Fig. 2:**
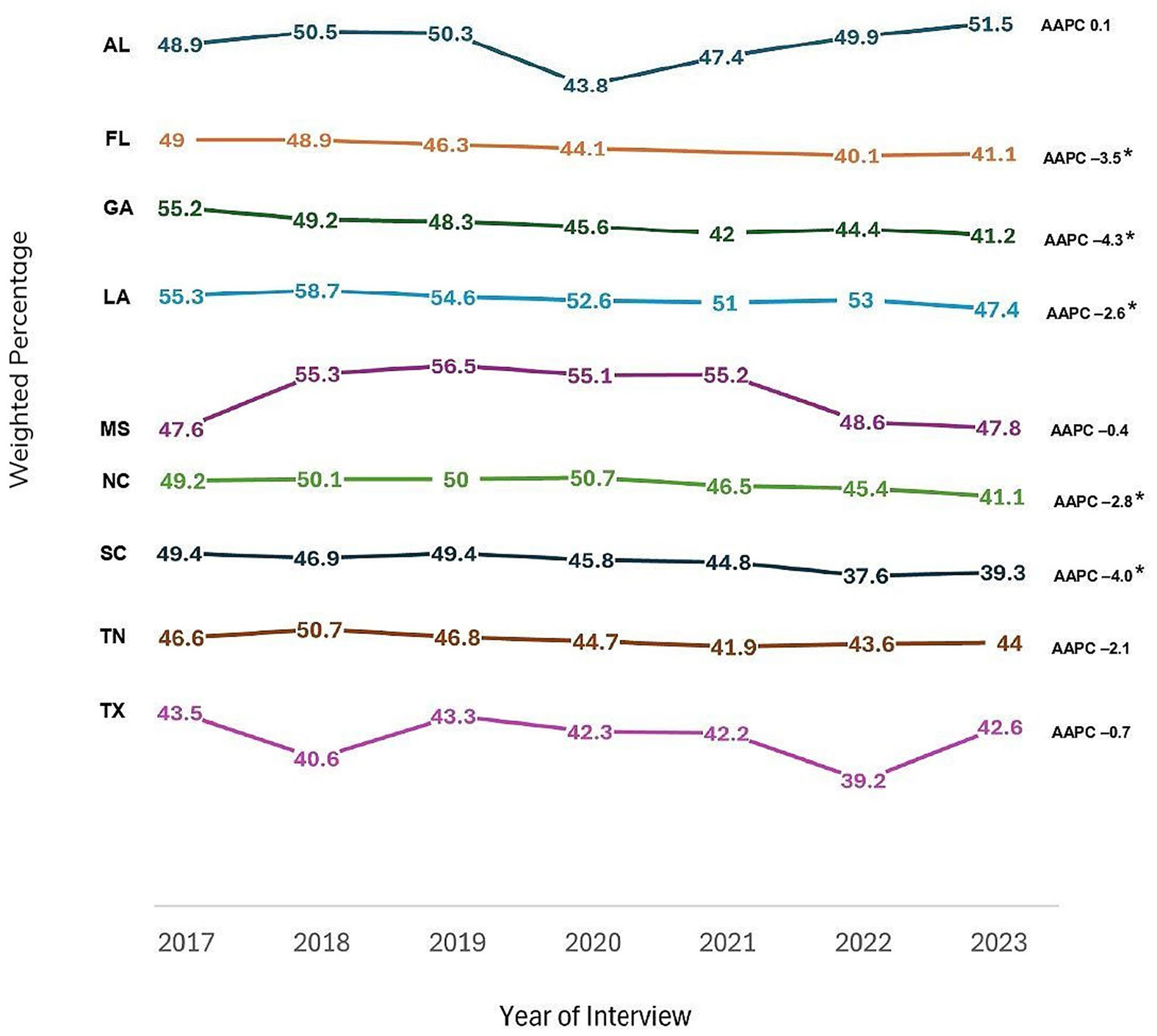
Trends in HIV testing in the past 12 months by state among adults in the deep south, behavioral risk factor surveillance system, 2017–2023. The Deep South is defined as Alabama [AL], Florida [FL], Georgia [GA], Louisiana [LA], Mississippi [MS], North Carolina [NC], South Carolina [SC], Tennessee [TN], and Texas [TX]. *Denotes statistical significance (Average Annual Percent Change [AAPC] 95% confidence interval does not include 1)

**Fig. 3:**
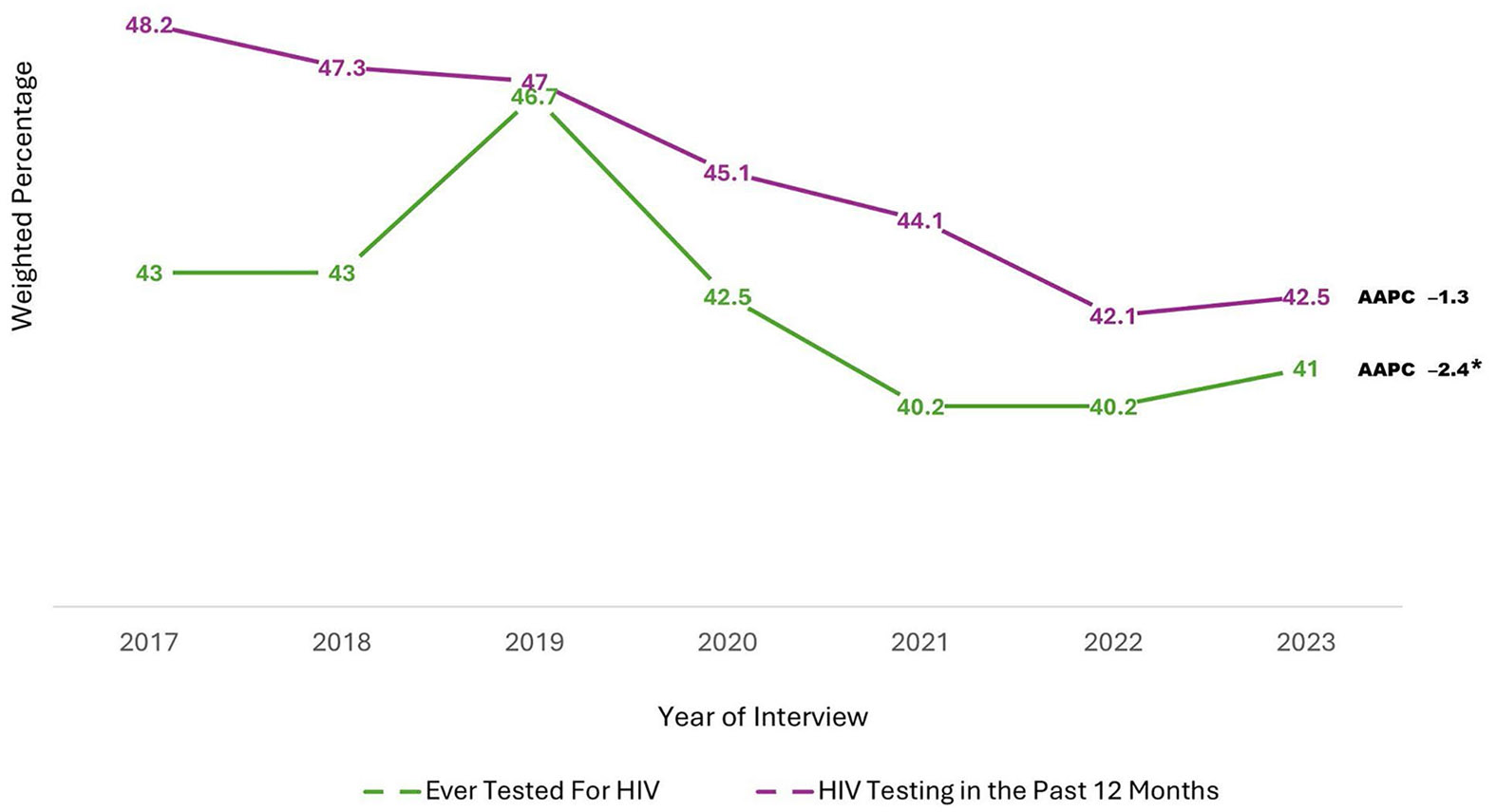
Trends in HIV testing among adults in the deep south: behavioral risk factor surveillance system, 2017–2023. The Deep South is defined as Alabama [AL], Florida [FL], Georgia [GA], Louisiana [LA], Mississippi [MS], North Carolina [NC], South Carolina [SC], Tennessee [TN], and Texas [TX]. *Denotes statistical significance (Average Annual Percent Change [AAPC] 95% confidence interval does not include 1)

Table 3 presents the weighted percentages of HIV testing in the past 12 months by year of interview, along with the AAPCs and corresponding 95% CIs of each variable. Figure 2 presents a graphical depiction of overall trends in HIV testing in the past 12 months by state among adults in the Deep South between 2017 and 2023. Additionally, Fig. 3 visually presents overall trends in ever been tested for HIV and HIV testing in the past 12 months among adults in the Deep South between 2017 and 2023.

## Discussion

This study examined trends in HIV testing among adults in the Deep South using 2017–2023 BRFSS data. Overall, the results point to decreasing HIV testing rates across Deep South states and sociodemographic factors. Despite ongoing public health efforts by the CDC to prevent and diagnose new HIV infections early [31], these decreasing trends indicate challenges in achieving optimal HIV testing rates in the Deep South region.

Our findings suggest that the overall percentage of ever been tested for HIV among respondents declined over the study period, however, this decrease was not statistically significant. Our results also suggest an overall significant decrease in the percentage of HIV testing in the past 12 months among adults, implying that fewer adults are being tested for HIV, either routinely or at any time, during the past year. Annual testing is imperative for the prevention and early diagnosis of HIV, particularly in a region such as the Deep South where the HIV prevalence is high [8].

Prior to our study period, national trends highlighted both an increase and decrease in HIV testing prevalence. According to the CDC [32], the National Health and Nutrition Examination Survey (NHANES) showed the percentage of adults who had ever been tested for HIV decreased from 42.5% in the 1999–2000 survey to 38.1% in the 2001–2002 survey, before rising again to 43.1% in the 2009–2010 survey. Additionally, the National Health Interview Survey data showed that the percentage of adults who had ever been tested for HIV increased significantly from 36.6% in 2000 to 45.0% in 2010, though trends fluctuated over time based on NHANES data for the same period [32]. Furthermore, between 2016 and 2017, Pitasi et al. [33] reported that 38.9% of U.S. adults aged ≥ 18 years had ever been tested for HIV, with only 29.2% of individuals at higher risk for HIV being tested in the past year. Pitasi et al. [33] attributes these suboptimal testing rates to missed opportunities to fully implement HIV screening recommendations and the variability in testing practices across jurisdictions. In the South specifically, studies highlight lack of awareness of HIV status and non-expansion of Medicaid as reasons for suboptimal HIV testing rates in this region [34, 35].

Our results also identified subpopulations that experienced significant declines in testing rates, illustrating potential disparities in HIV testing. For example, the percentage of ever been tested for HIV decreased significantly in North Carolina, among adults who were 25–44 years old, NH Black, NH multiracial, and identifying as lesbian or gay. These trends are particularly concerning given that studies show that certain populations including younger adults, Blacks/African Americans, and sexual minorities are at an increased risk of contracting HIV [2, 36, 37]. For instance, HIV.gov [2] highlighted that individuals aged 13 to 34 made up 60% of the estimated new HIV infections in 2022. Additionally, the CDC [38] noted that in 2021, Black people represented 12% of the U.S. population but comprised 40% of the estimated 32,100 new HIV infections that year. In the same year, Black gay and bisexual men were the most impacted group, comprising 37% of the estimated new infections among all gay and bisexual men [36]. In 2022, men who had sex with men made up 67% of new HIV diagnoses across the United States, including six territories and freely associated states [38]. Yet, our findings reveal a significant decrease in HIV testing among these at-risk populations, which could be attributed to several barriers such as limited access to healthcare, stigma, confidentiality concerns, negative treatment by healthcare staff, low perceived risk, and disruptions from the COVID-19 pandemic, among others [16, 34].

Furthermore, results showed a significant decrease in HIV testing within the past 12 months in FL, GA, LA, NC, and SC, as well as among various demographic groups, from 2017 to 2023. For example, there was a significant decline in testing within the past 12 months across all adult age groups except 35–44, as well as among NH White, NH Black, male, female, and heterosexual adults, among others. These findings suggest that declines in recent HIV testing are not confined to any single group but are widespread across different demographics. The decrease among males and females, and once again, among non-Hispanic Black adults, is particularly concerning given the disproportionate burden of HIV in these communities [39, 40]. The Kaiser Family Foundation [41] revealed that the rate per 100,000 of new HIV diagnosis among Black adults and adolescents (41.6) was approximately eight times higher than that of White individuals (5.3) and double the rate for Latinos (23.4) in 2022. Additionally, Black men had the highest rate across all racial/ethnic and gender groups at 66.3, while Black women (19.2) had the highest rate among women [41].

The COVID-19 pandemic may have played a role in the overall decline of HIV testing rates during this period, as healthcare systems were overwhelmed, and many non-emergency services, including HIV testing, were disrupted [42]. Additionally, several shelter-in-place orders across the U.S., beginning as early as March 2020, restricted the movement of persons from place-to-place. Moreover, the fear of exposure to COVID-19 in healthcare settings may have further reduced access to testing during this period, exacerbating the decline. Nosyk et al. [43] reported that HIV testing in the United States dropped significantly due to the COVID-19 pandemic, with the CDC recording 1,338,665 tests in 2020—a 44% decrease from 2019 and a 56% decrease from 2015, when 3,026,074 tests were conducted under CDC funding. Similarly, DiNenno et al. [44] noted that the pandemic disrupted healthcare services, leading to a 17% reduction in new HIV diagnoses reported to the CDC from 2019 to 2020, alongside a notable decline in HIV testing, including among priority populations within CDC-funded jurisdictions.

Despite these downward trends, we noted some positive trends during the study period. For example, in Alabama, there was a slight increase in HIV testing within the past 12 months, although there was a slight decreased trend in ever been tested for HIV in the state. Similarly, among adults earning an annual income of $50,000 or more, there was a slight decline in the rate of ever having been tested for HIV, but a slight increase in the percentage of those who tested in the past 12 months. These findings suggest that while fewer individuals in certain groups may have ever been tested for HIV, recent testing initiatives may have had some success in encouraging those already aware of their risk to continue regular testing.

While several trends in HIV testing rates among adults in the Deep South from 2017 to 2023 indicate significant declines, several other trends were not statistically significant. For instance, both males and females and Hispanic adults exhibited decreasing trends in ever having been tested for HIV, while non-Hispanic White adults showed an increase, though these trends were not significant. In terms of HIV testing within the past 12 months, non-significant declines were observed among adults aged 35–44, self-employed individuals, and those earning $35,000 or more, just to name a few. The lack of statistical significance in these trends may be attributed to factors such as variations in healthcare access, shifts in public health priorities, or differences in risk perception across subpopulations [34]. These findings may also suggest that testing rates may be stagnating or slightly declining among some subpopulations, which could have long-term implications for HIV prevention efforts.

These mixed results in HIV testing rates underscore the complexity of HIV testing behaviors. Similar to our findings, Ansa et al. [20], using BRFSS data from Georgia (2011–2015), reported slight declines in ever testing for HIV among young adults aged 18–24, as well as among males and females. Patel et al. [21], utilizing national BRFSS data from 2011 to 2017, also observed significant declines in HIV testing within the past 12 months among adults aged 34 years or younger. In contrast to our findings, Patel et al. [21] reported significant increases in ever testing for HIV and HIV testing within the past 12 months overall, as well as significant increases in ever testing for HIV among NH White, Hispanic, male, and female adults.

The significant decreases in testing among certain high-risk groups, such as young adults and racial and sexual minorities, highlight the urgency for tailored public health initiatives that specifically address the barriers faced by these populations such as limited access to healthcare, stigma, and mistrust, among others [16, 34]. Meanwhile, the observed increases in testing in Alabama, for example, may suggest that localized efforts may be effective in some contexts. Additionally, increased access to PrEP may have positively influenced HIV testing patterns, as PrEP uptake and ongoing PrEP use necessitates routine testing—every three months for oral PrEP and every two months for injectable PrEP, according to CDC guidelines [12, 45]. Public health programs should focus on reducing barriers to testing, including stigma and limited healthcare access, expanding HIV testing and PrEP programs and access, while also tailoring interventions to meet the specific needs of the most affected communities. This might include expanding community-based testing, increasing PrEP and testing awareness campaigns, and addressing the underlying social determinants of health, such as poverty and lack of insurance [46], which could help improve access to testing services and reduce the disparities.

## Strengths and Limitations

The strengths of this study include the large, diverse sample size from the BRFSS and its sampling design, which supports generalizability of the findings. Additionally, the use of weighted data allows for robust statistical analysis that accounts for sampling variability and population differences. However, BRFSS relies on self-reported data, which may overestimate or underestimate HIV testing trends. Additionally, self-reported data may be subject to recall bias, which may have contributed to the observed higher percentage of HIV testing in the past 12 months compared to the percentage of individuals who have ever been tested for HIV. Individuals who were tested within the past year may be more likely to recall and report their testing behavior, whereas those tested further in the past may underreport their testing history. Another limitation of the BRFSS data is its inability to capture contextual factors that may play a role in testing behaviors, such as stigma, or public health policies that may vary by state. Lastly, this study did not adjust for potential confounders, including health insurance coverage, which may have influenced HIV testing behaviors. Future research should consider incorporating health insurance data to better account for its potential impact on testing trends.

## Conclusion

While there have been modest gains in HIV testing in certain areas and among some groups, the overall decline in testing rates, particularly among high-risk populations, calls for scaling HIV prevention and care using mobile clinics, outreach testing programs, and free testing programs to eliminate disparities in HIV prevention and care. Given that declines in HIV testing were largely observed during the COVID-19 pandemic, these trends should be revisited now that we are in the post-pandemic period.

HIV testing is a critical entry point to both HIV prevention and care, aligning with the DHHS’s commitment under the EHE plan to diagnose all individuals with HIV as early as possible [47]. Our findings highlight the need for targeted interventions to sustain and enhance HIV testing efforts in the Deep South.

## Figures and Tables

**Table 1:** Sociodemographic characteristics of the study population, behavioral risk factor surveillance system, 2017–2023

Characteristics	*n*	Pop. Size	%	[95% CI]
State				
Alabama	39,207	27,199,062	5.3	[5.3–5.4]
Florida	92,707	105,544,150	20.7	[20.5–20.8]
Georgia	57,454	57,887,737	11.3	[11.2–11.4]
Louisiana	35,531	25,012,102	4.9	[4.9–4.9]
Mississippi	35,253	15,954,436	3.1	[3.1–3.2]
North Carolina	33,306	57,980,328	11.4	[11.3–11.4]
South Carolina	63,446	28,623,069	5.6	[5.6–5.6]
Tennessee	37,605	37,872,004	7.4	[7.4–7.5]
Texas	81,930	154,689,709	30.3	[30.1–30.5]
Age group				
18–24	28,394	62,943,723	12.3	[12.1–12.5]
25–34	51,392	87,645,180	17.2	[16.9–17.4]
35–44	58,516	84,245,518	16.5	[16.3–16.7]
45–54	72,324	82,345,814	16.1	[16.0–16.3]
55–64	90,807	82,053,293	16.1	[15.9–16.3]
65 or older	175,006	111,529,069	21.8	[21.6–22.0]
Race/ethnicity				
NH White	312,692	274,810,498	55.1	[54.9–55.4]
NH Black	84,418	93,956,948	18.9	[18.6–19.1]
NH Other Race	15,217	22,788,955	4.6	[4.4–4.7]
NH Multiracial	8068	7,764,369	1.6	[1.5–1.6]
Hispanic	44,271	99,105,561	19.9	[19.6–20.2]
Sex				
Male	210,245	246,860,783	48.3	[48.1–48.6]
Female	266,020	263,713,286	51.7	[51.4–51.9]
Sexual orientation				
Lesbian or Gay	2184	2,730,701	1.6	[1.5–1.7]
Heterosexual	161,822	159,169,260	93.3	[93.0–93.6]
Bisexual	4126	6,183,453	3.6	[3.4–3.8]
Other	1917	2,524,356	1.5	[1.4–1.6]
Educational level				
< High school	45,283	71,862,263	14.1	[13.9–14.4]
High school	130,042	145,238,501	28.6	[28.3–28.8]
Some College	130,998	156,762,216	30.9	[30.6–31.1]
College Graduate	167,752	134,157,005	26.4	[26.2–26.6]
Income (USD)				
<$15k	38,645	38,185,899	9.9	[9.7–10.1]
$15k–<$25k	62,220	63,583,478	16.4	[16.2–16.7]
$25k–<$35k	44,851	47,318,578	12.2	[12.0–12.4]
$35k–<$50k	53,033	54,169,027	14.0	[13.8–14.2]
$50k or more	165,187	183,656,721	47.5	[47.1–47.8]
Employment status				
Employed for wages	178,364	229,683,524	46.1	[45.8–46.3]
Self-Employed	38,001	48,020,887	9.6	[9.5–9.8]
Unemployed	101,743	122,789,001	24.6	[24.4–24.9]
Retired	149,424	98,186,979	19.7	[19.5–19.9]
Marital status				
Not married	101,510	142,728,874	28.2	[28.0–28.5]
Married	215,392	239,234,875	47.3	[47.0–47.6]
Divorced	91,193	83,671,187	16.5	[16.3–16.8]
Widowed	64,104	39,981,780	7.9	[7.8–8.0]
Ever tested for HIV?				
Yes	153,260	184,943,237	42.4	[42.1–42.7]
No	257,073	251,226,188	57.6	[57.3–57.9]
Tested for HIV ≤ 12 months?				
HIV testing ≤ 12 months	44,330	62,890,820	45.3	[44.8–45.9]
HIV testing > 12 months	64,577	75,847,608	54.7	[54.1–55.2]

n—unweighted count; Pop. Size—weighted count (population size); %—weighted percentage; CI—confidence interval, NH—non-Hispanic, NH Other race—Asians, American Indians, and Native Hawaiians, USD—United States Dollar

**Table 2:** Weighted percentages and average annual percent change in ever been tested for HIV among adults in the deep South, behavioral risk factor surveillance system, 2017–2023

	2017 (*n* = 79079)		2018 (*n* = 74070)		2019 (*n* = 71181)		2020 (*n* = 63028)		2021 (*n* = 52894)		2022 (*n* = 71056)		2023 (*n* = 65131)		Total (*N* = 476439)	AAPC [95% CI]
							
Category	*n*	%	*n*	%	*n*	%	*n*	%	*n*	%	*n*	%	*n*	%	*N*	
**Overall**	24,817	43.0	24,560	43.0	24,087	46.7	20,553	42.5	16,604	40.2	21,398	40.2	21,241	41.0	153,260	−1.3 [−4.0, 1.6]
State																
Alabama	2022	40.2	2060	39.4	2307	44.6	1712	39.0	1359	37.2	1296	36.4	1386	38.1	12,142	−1.7 [−4.9, 1.6]
Florida	7994	47.0	5373	45.0	6060	50.7	4053	46.9	N/A	N/A	4125	44.1	4372	42.9	31,977	−1.4 [−4.4, 1.8]
Georgia	2152	47.5	3439	46.4	2621	50.2	3260	48.4	2666	44.7	2921	44.9	2691	44.1	19,750	−1.3 [−3.1, 0.6]
Louisiana	1403	40.3	1699	42.5	1688	48.0	1497	42.5	1642	42.7	1747	41.9	1960	44.8	11,636	0.5 [−2.6, 3.7]
Mississippi	1280	39.1	1984	41.0	1710	43.5	1836	37.6	1312	37.7	1373	38.1	1319	37.7	10,814	−1.5 [−4.2, 1.2]
North Carolina	1776	45.0	1854	45.7	1701	47.1	1985	39.4	1807	40.4	1559	39.4	1425	40.0	12,107	−3.0 [−5.7, −0.2]*
South Carolina	3021	37.6	2989	37.8	2204	42.1	1281	40.3	2779	40.1	2606	36.1	2962	38.3	17,842	−0.1 [−2.5, 2.4]
Tennessee	1635	37.7	1519	37.3	1885	42.6	1357	38.6	1457	37.7	1530	38.2	1756	38.8	11,139	−0.1 [−2.6, 2.6]
Texas	3534	41.0	3643	42.3	3911	44.8	3572	40.4	3582	39.6	4241	37.8	3370	40.1	25,853	−1.6 [−4.0, 0.8]
Age group																
18–24	1419	36.3	1383	34.9	1405	40.5	1042	29.8	812	29.6	999	26.7	1070	30.6	8130	−4.7 [−9.7, 0.7]
25–34	4256	59.0	4175	57.8	3899	62.4	3230	53.3	2488	50.0	2972	48.7	2974	50.5	23,994	−3.6 [−6.5, −0.6]*
35–44	4743	63.2	4912	62.2	4551	65.3	4162	58.8	3448	56.5	4267	57.2	4130	56.6	30,213	−2.3 [−4.0, −0.5]*
45–54	5271	52.0	5126	51.1	4947	56.2	4198	52.1	3695	50.9	4496	53.8	4436	52.6	32,169	0.1 [−1.8, 2.1]
55–64	5043	35.7	4812	37.4	4923	42.4	3905	40.3	3080	37.0	4269	40.0	4161	41.0	30,193	1.4 [−1.6, 4.5]
65 or older	4085	16.9	4152	18.6	4362	20.6	4016	22.9	3081	19.5	4395	20.2	4470	21.6	28,561	2.8 [−1.1, 6.9]
Race/ethnicity																
NH White	14,221	36.2	13,592	35.9	13,695	39.2	11,374	35.5	9105	34.7	12,210	33.7	11,959	35.0	86,156	1.3 [−3.6, 1.1]
NH Black	5950	62.4	6779	63.2	5849	65.9	5151	61.7	4455	56.7	5212	57.5	4854	55.1	38,250	−2.4 [−4.4, −0.4]*
NH Other Race	816	37.2	794	36.4	787	38.3	695	31.9	555	31.1	535	29.7	822	34.4	5004	−3.0 [−6.7, 0.8]
NH Multiracial	539	57.4	541	59.8	643	62.0	472	56.0	438	55.7	526	49.1	572	51.5	3731	−2.9 [−5.5, −0.1]*
Hispanic	2725	45.0	2362	44.7	2638	52.0	2454	44.5	1702	39.8	2304	42.8	2356	43.6	16,541	−1.6 [−5.3, 2.3]
Sex																
Male	10,842	41.9	10,705	41.4	10,428	44.8	9183	40.8	7612	39.0	9870	38.9	9823	39.9	68,463	−1.5 [−3.6, 0.5]
Female	13,963	44.0	13,827	44.4	13,659	48.6	11,370	44.0	8992	41.4	11,528	41.5	11,418	42.0	84,757	−1.5 [−4.2, 1.2]
Sexual orientation																
Lesbian or Gay	491	72.2	134	57.2	182	65.9	98	54.1	94	48.4	105	49.2	114	51.4	1218	−6.7 [−10.3, 3.0]*
Heterosexual	17,393	42.3	8361	42.6	9944	47.4	5189	43.4	4844	42.0	4546	41.4	4693	41.6	54,970	−0.5 [−3.3, 2.3]
Bisexual	467	55.9	363	63.1	429	62.6	245	58.7	236	48.5	265	44.6	264	54.0	2269	−3.2 [−7.9, 1.7]
Other	60	30.2	85	41.3	119	43.4	104	45.2	83	32.7	89	35.4	95	37.8	635	−0.5 [−8.3, 8.0]
Education level																
< High School	2501	40.1	2394	36.5	2457	43.8	1980	39.8	1367	38.0	1550	36.8	1606	37.9	13,855	−1.1 [−4.3, 2.3]
High School	6303	39.8	6241	40.1	6205	45.1	5320	39.7	3867	36.5	4965	36.7	4955	37.7	37,856	−2.1 [−5.3, 1.3]
Some College	7454	45.7	7436	46.9	7296	48.8	6185	45.8	4949	43.6	6527	43.5	6254	44.9	46,101	−1.2 [−3.0, 0.6]
College Grad	8501	45.2	8428	45.2	8066	47.8	7003	42.9	6357	41.5	8281	41.9	8343	41.7	54,979	−2.1 [−4.0, −0.0]*
Annual income (USD)																
<$15k	3141	47.9	2846	46.8	2893	52.4	2318	47.9	1346	48.7	1572	45.3	1481	47.1	15,597	−0.6 [−3.1, 2.0]
$15k–<$25k	4599	45.9	4250	46.6	4113	49.8	3402	45.5	1831	43.4	2269	43.3	1961	44.4	22,425	−1.3 [−3.7, 1.3]
$25k–<$35k	2371	45.8	2395	45.6	2157	49.7	1894	46.0	1939	40.9	2433	42.8	2197	41.6	15,386	−2.1 [−4.7, 0.5]
$35k–<$50k	2889	44.4	2888	44.6	2720	47.1	2355	44.0	1945	43.7	2405	41.7	2503	42.1	17,705	−1.4 [−3.0, 0.2]
$50k or more	8783	43.3	8985	43.5	8906	47.5	7719	43.0	6992	41.3	5105	41.1	10,007	42.9	56,497	−1.0 [−3.5, 1.5]
Employment status																
Employed	11,518	50.4	11,661	49.6	11,001	53.5	9394	47.4	7999	44.7	10,132	45.9	9948	45.9	71,653	−2.2 [−4.5, 0.2]
Self-employed	2195	48.2	2146	46.0	2202	50.6	1756	47.2	1485	45.5	2028	45.1	2018	47.7	13,830	−0.7 [−2.7, 1.3]
Unemployed	7062	44.8	6617	45.3	6655	51.8	5613	45.7	4099	43.6	4957	43.4	4964	44.8	39,967	−1.0 [−4.4, 2.5]
Retired	3830	19.5	3917	21.6	4039	22.6	3618	24.3	2880	22.1	4056	22.2	4073	23.5	26,413	2.0 [−0.8, 4.8]
Marital status																
Not married	7344	50.6	1632	22.1	7365	55.2	6166	46.6	4926	44.3	6232	43.6	6315	44.6	39,980	−2.6 [−9.4, 4.8]
Married	10,998	39.2	7635	50.1	10,350	42.7	8975	39.6	7536	37.3	9782	37.7	9607	38.4	64,883	−2.7 [−6.8, 1.7]
Divorced	4636	51.9	10,616	39.0	4373	55.6	3692	53.8	2816	52.5	3707	53.9	3607	53.2	33,447	3.6 [−3.4, 11.0]
Widowed	1702	22.6	4519	53.5	1820	25.1	1581	27.5	1180	25.0	1492	22.5	1547	26.0	13,841	−11.8 [27.6, 7.3]

Acronym: n—unweighted count, N—total unweighted count, %—weighted percentage, AAPC—Average Annual Percentage Change, CI—confidence interval, NH—non-Hispanic, NH Other race—Asians, American Indians, and Native Hawaiians, N/A—data not available, USD—United States Dollar, *—significant finding

**Table 3:** Weighted percentages and average annual percent change in HIV testing in the past 12 months among adults in the deep South, behavioral risk factor surveillance system, 2017–2023

	2017 (*n* = 79079)		2018 (*n* = 74070)		2019 (*n* = 71181)		2020 (*n* = 63028)		2021 (*n* = 52894)		2022 (*n* = 71056)		2023 (*n* = 65131)		Total (*N* = 476439)	AAPC [95% CI]
							
Category	*n*	%	*n*	%	*n*	%	*n*	%	*n*	%	*n*	%	*n*	%	*N*	
**Overall**	8002	48.2	7647	47.3	7607	47.0	5700	45.1	4462	44.1	5429	42.1	5483	42.5	44,330	−2.4 [−4.0, −0.7]*
State																
Alabama	625	48.9	626	50.5	686	50.3	408	43.8	336	47.4	322	49.9	384	51.5	3387	0.1 [−2.5, 2.8]
Florida	2496	49.0	1637	48.9	1848	46.3	1052	44.1	N/A	N/A	992	40.1	1018	41.1	9043	−3.5 [4.8, −2.2]*
Georgia	815	55.2	1169	49.2	978	48.3	979	45.6	718	42.0	791	44.4	758	41.2	6208	−4.3 [−6.2, −2.5]*
Louisiana	508	55.3	620	58.7	637	54.6	454	52.6	475	51.0	503	53.0	543	47.4	3740	−2.6 [−4.5, −0.7]*
Mississippi	426	47.6	631	55.3	554	56.5	530	55.1	375	55.2	370	48.6	395	47.8	3281	−0.4 [−4.2, 3.5]
North Carolina	596	49.2	566	50.1	533	50.0	558	50.7	472	46.5	384	45.4	329	41.1	3438	−2.8 [−4.8, −0.9]*
South Carolina	996	49.4	889	46.9	657	49.4	337	45.8	719	44.8	594	37.6	703	39.3	4895	−4.0 [−6.3, −1.7]*
Tennessee	464	46.6	443	50.7	526	46.8	340	44.7	341	41.9	356	43.6	427	44.0	2897	−2.1 [−4.3, 0.2]
Texas	1076	43.5	1066	40.6	1188	43.3	1042	42.3	1026	42.2	1117	39.2	926	42.6	7441	−0.7 [−2.6, 1.2]
Age group																
18–24	946	78.2	887	77.4	959	76.6	652	71.8	473	66.7	554	67.4	591	68.3	5062	−2.9 [−4.1, −1.6]*
25–34	2079	55.7	2045	57.4	1901	55.2	1479	54.6	1086	54.5	1261	50.4	1272	52.4	11,123	−1.6 [−2.8, −0.3]*
35–44	1642	42.0	1644	41.1	1593	44.0	1331	45.3	1047	40.7	1322	44.8	1326	43.3	9905	−0.7 [−1.2, 2.6]
45–54	1417	38.3	1354	35.3	1262	33.9	938	33.2	884	35.3	943	31.4	1005	31.7	7803	−2.7 [−4.6, −0.8]*
55–64	1161	36.1	1049	36.3	1101	35.1	711	30.2	547	30.9	734	30.3	711	31.7	6014	−3.2 [−5.3, −1.0]*
65 or older	757	38.7	668	31.7	791	33.7	589	33.2	425	32.0	615	28.7	578	25.2	4423	−5.1 [−8.3, −1.8]*
Race/ethnicity																
NH White	3562	38.4	3303	37.7	3432	37.1	2436	35.0	1887	33.4	2412	32.0	2452	33.6	19,484	−3.0 [−4.3, −1.7]*
NH Black	2745	65.5	2894	64.1	2524	60.4	1962	59.9	1596	57.6	1760	53.3	1679	53.9	15,160	−3.5 [−4.3, −2.7]*
NH Other Race	271	50.6	270	41.6	298	52.4	208	43.9	175	47.0	163	49.5	207	43.9	1592	−0.6 [−5.2, 4.3]
NH Multiracial	195	46.7	202	54.4	231	46.9	166	47.4	148	53.3	169	43.4	170	51.0	1281	−0.3 [−4.6, 4.2]
Hispanic	1058	47.8	851	45.9	958	51.1	830	46.3	566	47.0	776	47.6	781	45.2	5820	−0.6 [−2.6, 1.5]
Sex																
Male	3645	48.7	3537	49.3	3471	48.6	2620	45.5	2037	43.5	2562	42.4	2607	44.0	20,479	−2.6 [−4.0, −1.2]*
Female	4354	47.7	4101	45.5	4136	45.6	3080	44.8	2425	44.6	2867	41.9	2876	41.2	23,839	−2.2 [−3.0, −1.5]*
Sexual orientation																
Lesbian or Gay	226	60.7	46	44.7	62	50.1	28	40.6	33	55.3	32	48.0	29	59.6	456	−2.5 [−7.9, 3.3]
Heterosexual	5367	46.6	2347	43.4	2873	42.3	1424	44.1	1293	42.7	1121	40.0	1160	39.2	15,585	−2.6 [−3.9, −1.2]*
Bisexual	220	58.7	158	56.1	205	57.4	114	61.7	104	56.1	108	52.5	103	52.6	1012	−1.6 [−3.9, 0.7]
Other	25	71.3	25	29.8	47	62.4	40	54.4	23	40.8	23	47.5	33	39.8	216	−7.1 [−17.5, 4.7]
Education level																
< High School	876	52.3	705	49.4	775	53.4	530	43.3	334	41.7	398	47.9	343	35.5	3961	−4.4 [−8.7, 0.2]
High School	2231	50.0	2110	51.7	2134	51.2	1543	47.9	1162	49.1	1363	46.4	1396	48.5	11,939	−1.3 [−2.7, 0.1]
Some College	2493	51.3	2445	49.3	2374	45.9	1744	44.6	1338	44.7	1663	41.3	1669	44.2	13,726	−3.0 [−4.7, −1.3]*
College Grad	2389	40.6	2373	39.7	2308	41.4	1863	43.9	1617	39.9	1988	37.3	2057	37.8	14,595	−1.3 [−3.7, 1.1]
Annual income (USD)																
<$15k	1167	57.2	1023	58.3	1060	55.1	747	51.2	416	48.5	439	50.5	441	51.2	5293	−2.7 [−4.5, −0.9]*
$15k–<$25k	1742	53.9	1551	53.2	1466	53.1	1050	49.9	546	50.4	656	47.3	541	42.0	7552	−3.0 [−4.6, −1.4]*
$25k–<$35k	875	56.8	798	49.6	736	49.2	571	46.3	572	47.1	693	46.1	663	45.7	4908	−3.2 [−5.2, −1.2]*
$35k–<$50k	955	48.3	969	48.8	917	46.2	663	44.1	576	49.1	654	43.4	725	48.2	5459	−0.7 [−3.1, 1.8]
$50k or more	2319	38.5	2418	40.0	2441	40.9	1970	41.1	1767	39.0	1326	42.2	2456	39.7	14,697	0.5 [−1.0, 2.0]
Employment status																
Employed	4007	47.7	4056	48.8	3811	48.1	2908	45.9	2406	44.8	2844	42.1	2924	44.5	22,956	−2.1 [−3.5, −0.6]*
Self-employed	629	40.8	598	38.8	662	39.3	464	46.2	361	36.4	495	37.3	508	39.3	3717	−0.9 [−4.6, 2.9]
Unemployed	2492	53.1	2212	50.6	2303	50.0	1716	47.0	1211	48.9	1414	48.0	1403	45.3	12,751	−2.2 [−3.3, −1.1]*
Retired	786	41.6	697	35.0	760	34.4	555	31.0	439	32.1	612	28.7	584	28.4	4433	−5.8 [−8.0, −3.5]*
Marital status																
Not married	3447	61.3	347	39.6	3448	61.5	2628	60.2	1949	56.7	2354	54.5	2486	56.0	16,659	−1.8 [−4.8, 1.3]
Married	2651	37.4	3461	61.9	2399	35.3	1818	34.5	1549	34.2	1890	32.7	1828	31.5	15,596	−8.7 [−19.5, 3.6]
Divorced	1445	48.4	2433	35.4	1318	44.9	931	40.3	712	42.1	877	40.1	882	42.4	8598	−0.3 [−6.3, 6.0]
Widowed	421	41.2	1359	44.7	387	36.7	277	30.0	205	39.9	256	36.2	242	32.8	3147	−5.2 [−11.0, 1.1]

Acronym: n—unweighted count, N—total unweighted count, %—weighted percentage, AAPC—Average Annual Percentage Change, CI—confidence interval, NH—non-Hispanic, NH Other race—Asians, American Indians, and Native Hawaiians, N/A—data not available, USD—United States Dollar, *—significant finding
